# *miR-941* as a promising biomarker for acute coronary syndrome

**DOI:** 10.1186/s12872-017-0653-8

**Published:** 2017-08-22

**Authors:** Ruina Bai, Qiaoning Yang, Ruixi Xi, Lizhi Li, Dazhuo Shi, Keji Chen

**Affiliations:** Xiyuan Hospital, China Academy of Chinese Medical Sciences, 1 Xiyuan Caochang, Haidian district, Beijing, China

**Keywords:** microRNA, Stable angina, Acute ST-segment elevation myocardial infarction, Acute coronary artery disease

## Abstract

**Background:**

Circulating miRNAs can function as biomarkers for diagnosis, treatment, and prevention of diseases. However, it is unclear whether miRNAs can be used as biomarkers for acute coronary syndrome (ACS). To this end, we applied gene chip technology to analyze miRNA expression in patients with stable angina (SA), non-ST elevation ACS (NSTE-ACS), and ST-segment elevation myocardial infarction (STEMI).

**Methods:**

We enrolled patients with chest pain who underwent diagnostic coronary angiography, including five patients each with SA, NSTE-ACS, or STEMI, and five controls without coronary artery disease (CAD) but with three or more risk factors. After microarray analysis, differential miRNA expression was confirmed by quantitative real-time reverse transcription polymerase chain reaction (qRT-PCR).

**Results:**

Compared with those in patients with STEMI, differentially expressed microRNAs in controls and patients with SA or NSTE-ACS were involved in inflammation, protein phosphorylation, and cell adhesion. Pathway analysis showed that differentially expressed miRNAs were related to the mitogen-activated protein kinase signaling, calcium ion pathways, and cell adhesion pathways. Compared with their expression levels in patients with STEMI, *miR-941*, *miR-363-3p*, and *miR-182-5p* were significantly up-regulated (fold-change: 2.0 or more, *P* < 0.05) in controls and patients with SA or NSTE-ACS. Further, qRT-PCR showed that plasma *miR-941* level was elevated in patients with NSTE-ACS or STEMI as compared with that in patients without CAD (fold-change: 1.65 and 2.28, respectively; *P* < 0.05). Additionally, *miR-941* expression was significantly elevated in the STEMI group compared with that in the SA (*P* < 0.01) and NSTE-ACS groups (*P* < 0.05). Similarly, *miR-941* expression was higher in patients with ACS (NSTE-ACS or STEMI) than in patients without ACS (without CAD or with SA; *P* < 0.01). There were no significant differences in *miR-182-5p* and *miR-363-3p* expression. The areas under the receiver operating characteristic curves were 0.896, 0.808, and 0.781 for patients in the control, SA, and NSTE-ACS groups, respectively, compared with that for patients with STEMI; that for the ACS group compared with the non-ACS group was 0.734.

**Conclusion:**

*miR-941* expression was relatively higher in patients with ACS and STEMI. Thus, *miR-941* may be a potential biomarker of ACS or STEMI.

**Electronic supplementary material:**

The online version of this article (doi:10.1186/s12872-017-0653-8) contains supplementary material, which is available to authorized users.

## Background

Acute coronary syndrome (ACS) is a major cause of death and disability [1]. Rapid antiplatelet therapy and revascularization could prevent myocardial ischemia and reduce the incidence of cardiovascular events [2]. Thus, the early diagnosis of non-ST elevation ACS (NSTE-ACS) and ST-segment elevation myocardial infarction (STEMI) is essential for improved prognoses. Currently, the clinical diagnosis of ACS relies on assessment of symptoms, ischemia changes in electrocardiogram (ECG), and changes in troponin [2]. However, in elderly individuals and patients with diabetes, typical symptoms are not always observed. Moreover, changes in ECG can be easily influenced by left bundle branch blockage and chronic myocardial infarction. To a certain extent, unstable angina pectoris (UA), non-STEMI (NSTEMI), and STEMI reflect the pathological progress of ACS. Therefore, identification of novel biomarkers may facilitate the early diagnosis of ACS, particularly in patients with atypical symptoms of ACS.

MicroRNAs (miRNAs) are endogenous, small (22–24-nucleotide) noncoding RNA molecules that regulate the expression of mRNA by combining with the 3′- untranslated region (3′-UTR), subsequently triggering the degradation of mRNA or having negative effects on transcription [3]. miRNAs can regulate nearly 60% of coding genes to exert their biological functions [4, 5]. Recently, many studies have shown that miRNAs can regulate endothelial dysfunction, inflammation, cell autophagy, platelet activation, and aggregation [6–9]. Moreover, miRNA expression can affect the stability of atherosclerotic plaques [10]. miRNAs possess tissue-specific expression and can be secreted into blood or urine.miRNAs of circulartory system can be used as biomarkers of diagnosis, treatment, and prevention of diseases, such as coronary heart disease [11–14]. However, it is still unclear whether miRNAs can be used as biomarkers of ACS and further evaluate the severity of ACS [15].

Therefore, in this study, we applied gene chip technology to analyze the expression of miRNAs in patients with stable angina (SA), NSTE-ACS, and STEMI. We then performed quantitative real-time reverse transcription polymerase chain reaction (qRT-PCR) to determine whether miRNAs could be used as biomarker for the diagnosis of ACS.

## Methods

### Patients and study design

**Fig. 1:**
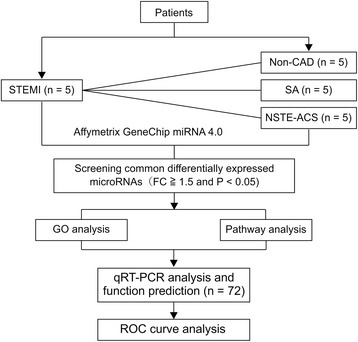
Schematic of the study design

A total of 72 patients with chest pain who underwent diagnostic coronary angiography in Xiyuan Hospital, affiliated hospital of China Academy of Chinese Medical Sciences were enrolled from March 2015 to July 2015. The patients were divided into four groups as follows: 18 patients with STEMI, 18 patients NSTE-ACS, 20 patients with SA, and 16 patients without CAD. Patients in the control group did not have coronary stenosis as confirmed by coronary angiography, but had three or more risk factors for coronary heart disease (see schematic of the study in Fig. 1). Five cases were randomly selected from each group for analysis of gene expression profiles, using an Affymetrix GeneChip miRNA4.0. The differential expression of miRNAs between groups was analyzed according to the following criteria: fold-change (FC), ≥ 1.5 and *P* < 0.05. qRT-PCR was applied to verify the differential expression of miRNAs (FC, ≥ 2; *P* < 0.05). This study was registered in the Chinese Clinical Trial Registry (no. ChiCTR-IPR- 15006336). The study was performed according to the guidelines of the Declaration of Helsinki and was approved by the Xiyuan Hospital Ethics Committee.

### Diagnostic criteria

In this study, patients with coronary heart disease were classified as having SA or ACS, including STEMI, NSTE-ACS, and UA. Diagnostic criteria was refered to the 2014 ACC/AHA/AATS/PCNA/SCAI/STS Focused Update of the Guideline for the Diagnosis and Management of Patients With Stable Ischemic Heart Disease [16], 2014 AHA/ACC Guideline for the Management of Patients with Non-ST-Elevation Acute Coronary Syndromes [2], and 2013 ACCF/AHA Guideline for the Management of ST-elevation Myocardial Infarction [17]. In addition, all patients presented stenosis (≥ 50% in at least one main coronary artery) as confirmed by coronary angiography [18].

### Inclusion and exclusion criteria

The inclusion criteria were as follows: patients who met the diagnostic criteria, were 35–75 years old, and provided informed consent. The exclusion criteria were as follows: Patients with combined diseases, such as cardiomyopathy, valvular heart disease, severe arrhythmia, heart failure, and other accompanying diseases; patients encountered challenges with data collection, such as religious or language barriers; patients were pregnant or lactating and participating in other clinical studies.

### Blood collection and storage

Venous blood samples were collected via antecubital venipuncture from each subject within 3–5 h of the onset of symptoms but before arteriography. Whole blood samples (2 mL) were collected directly into EDTA-containing tubes (BD, Franklin Lakes, NJ, USA), and three volumes of red blood cell lysis buffer (NH4CL2009; Haoyang, Tianjin, China) was added to obtain leukocytes, which were isolated within 2 h by centrifugation at 3000 rpm for 5 min at 4 °C to remove other blood elements. Next, 1 mL TRIzol (15596–026; Invitrogen Life Technologies) was added, and samples were then transferred to RNase/Dnase-free tubes and stored at −80 °C.

### RNA isolation and preparation

After collection of all samples, total RNA in leukocytes was isolated using a miRVana RNA Isolation Kit (p/n AM1556; Applied Biosystems, Foster City, CA, USA) according to the manufacturer’s specifications. Samples were then subjected to on-column DNase I treatment with RNase-free Dnase (#79254; Qiagen, Valencia, CA, USA). RNA quantity and quality were determined using a NanoDrop 2000 spectrophotometer (Thermo Scientific, USA) and an Agilent 2100 bioanalyzer. The RNA integrity was evaluated by agarose gel electrophoresis with ethidium bromide staining.

### miRNA array analysis

Microarray analysis of gene expression was carried out using ELOSA QC Assays prior to array hybridization. Sample labeling, microarray hybridization, and washing were performed based on the manufacturer’s standard protocols. Briefly, total RNA was modified with poly A tails and then labeled with biotin. Next, the labeled RNAs were hybridized onto the microarray. Slides were washed and stained, and the arrays were scanned using an Affymetrix Scanner 3000 (Affymetrix).

miRNA microarray analysis was performed to determine differential expression of blood-borne miRNAs among (i) non-CAD individuals and patients with STEMI (*n* = 5), (ii) patients with SA and STEMI (*n* = 5), and (iii) patients with NSTE-ACS and STEMI. Bioinformatic determination of downstream predicted targets for candidate miRNAs was performed as described previously by Selbach et al. [19].

### Reverse transcription quantitative real-time polymerase chain reaction (RT-qPCR) analysis

Quantification was performed through two-step reaction process: reverse transcription (RT) and qPCR. Each RT reaction consisted of 1 μg RNA, 4 μL miScript HiSpec Buffer, 2 μL Nucleics Mix, and 2 μL miScript Reverse Transcriptase Mix (Qiagen, Germany), in a total volume of 20 μl. Reactions were performed in a GeneAmp PCR System 9700 (Applied Biosystems, USA) for 60 min at 37 °C, followed by heat inactivation of the reverse transcriptase for 5 min at 95 °C. The 20-μL reaction mix was then diluted 5-fold in nuclease-free water and stored at −20 °C.

Real-time PCR was performed using a LightCycler 480II Real-time PCR Instrument (Roche, Switzerland) with 10 μL of the reaction mixture including 1 μL cDNA, 5 μL 2× LightCycler 480 SYBR Green I Master (Roche), 0.2 μL universal primers (Qiagen), 0.2 μL miRNA-specific primer, and 3.6 μL nuclease-free water. Reactions were incubated in a 384-well optical plate (Roche) at 95 °C for 10 min, followed by 40 cycles of 95 °C for 10 s and 60 °C for 30 s. Triplicates were averaged to calculate the expression value for each sample. At the end of the PCR cycling, melt curve analysis was performed to validate the specific generation of the expected PCR product. miRNA-specific primer sequences were designed in the laboratory and synthesized by Generay Biotech (Generay, PRC) based on the miRNA sequences obtained from the miRBase database (Release 20.0) as follows: *hsa-miR-182-5p*, UUUGGCAAUGGUAGAACUCACACU; *hsa-miR-363-3p*, AAUUGCACGGUAUCCAUCUGUA; and *hsa-miR-941*, CACCCGGCUGUGUGCACAUGUGC).

The expression levels of miRNAs were normalized to U6 and were calculated by the 2^-ΔΔCt^ method [20].

### Statistical analysis

Affymetrix GeneChip Command Console software (version4.0, Affymetrix) was used to analyze array images to obtain raw data, and RMA normalization was then carried out. Next, Genespring software (version 12.5, Agilent Technologies) was used for subsequent data analysis. Differentially expressed miRNAs were then identified through fold changes, and *P*-values were calculated using t-tests. The threshold set for up- and downregulated genes was a fold change of 1.5 or more and a *P* value of less than 0.05. Target genes of differentially expressed miRNAs were the intersection predicted with three databases (Targetscan, PITA, and microRNAorg). Gene ontology (GO) analysis and KEGG analysis were applied to determine the roles of these target genes. Hierarchical clustering was performed to show distinguishable miRNA expression patterns among samples.

Paired and unpaired Student’s t tests were performed to compare data as appropriate. Values are expressed as means ± standard deviations (SDs). *P* values of less than 0.05 (two-sided) were considered significant.

## Results

### Patient clinicopathological information

Seventy-two patients (18 patients with STEMI, 18 patients with NSTE-ACS, 20 patients with SA, and 16 patients without CAD) were enrolled in this study. The clinicopathological characteristics of the patients are presented in Tables 1 and 2. There were no significant differences in clinical features among groups (*P* > 0.05).

**Table 1:** Baseline clinical characteristics of different patient groups used in microarray analysis

Variable	CAD (*n* = 15)			Without CAD (*n* = 5)
	SA (*n* = 5)	NSTE-ACS (*n* = 5)	STEMI (*n* = 5)	
Age (years, mean ± SD)	55.8 ± 4.15	57.4 ± 11.19	52.8 ± 10.56	52.4 ± 13.28
Sex (male, n)	5	5	5	5
Risk factors (n)				
Hypertension	4	4	2	3
Dyslipidemia	5	3	4	3
Active smoker	4	3	5	1
Medications (*n*)				
Antiplatelet agents	5	5	5	0
β-Blockers	4	3	3	0
CCB	2	1	1	1
ACEI/ARB	4	4	4	2
Statins	5	5	5	1

*Abbreviations*: *SA* stable angina, *NSTE-ACS* non-ST elevation acute coronary syndrome, *STEMI* ST elevation myocardial infarction, *DM* diabetes mellitus, *CCB* calcium channel blocker, *ACEI* angiotensin-converting enzyme inhibitor, *ARB* angiotensin receptor blocker

**Table 2:** Baseline clinical characteristics of different patient groups used in qRT-PCR analysis

Variable	CAD (*n* = 56)			Without CAD (*n* = 16)
	SA (*n* = 20)	NSTE-ACS (*n* = 18)	STEMI (*n* = 18)	
Age (years, mean ± SD)	55.8 ± 9.15	56.44 ± 8.78	53.50 ± 10.56	52.5 ± 13.50
Sex (male, n)	12	15	16	9
Risk factors (n)				
Hypertension	14	15	13	11
Dyslipidemia	12	13	11	8
DM	9	10	9	7
Active smoker	12	15	14	8
Medications (n)				
Antiplatelet agents	12	16	18	0
β-Blockers	13	11	9	9
Statins	18	16	12	8

*Abbreviations*: *SA* stable angina, *NSTE-ACS* non-ST elevation acute coronary syndrome, *STEMI* ST elevation myocardial infarction, *DM* diabetes mellitus, *CCB* calcium channel blocker, *ACEI* angiotensin-converting enzyme inhibitor, *ARB* angiotensin receptor blocker

### miRNA array analysis

The differential expression of miRNAs among the patient groups was determined by gene chip analysis. The results showed that there exist 13 differentially expressed miRNAs in patients with STEMI comparing with those in patients without CAD and patients with SA and NSTE-ACS. After that, we chose *miR-941*, *miR-363-3p*, and *miR-182-5p* (FC,≥ 2.0; *P* < 0.05) for further analysis by qRT-PCR (Table 3).

**Table 3:** Differentially expressed miRNAs in patients with ACS versus patients without CAD and patients with SA or NSTE-ACS in microarray analysis

miRNAs	STEMI versus without CAD			STEMI versus SA			STEMI versus NSTE-ACS		
	Fold change	*P* value	Regulation	Fold change	*P* value	Regulation	Fold change	*P* value	Regulation
*miR-941*	5.305	0.007	Up	4.544	0.003	Up	5.032	0.002	Up
*miR-182-5p*	2.331	0.013	Down	2.005	0.042	Down	2.121	0.024	Up
*miR-363-3p*	2.012	0.007	Down	2.016	0.018	Down	2.071	0.048	Up
*hsa-mir-941-1*	1.895	0.036	Up	2.051	0.001	Up	2.008	0.001	Up
*hsa-mir-941-2*	1.895	0.036	Up	2.051	0.001	Up	2.008	0.001	Up
*hsa-mir-941-3*	1.895	0.036	Up	2.051	0.001	Up	2.008	0.001	Up
*hsa-mir-941-4*	1.895	0.036	Up	2.051	0.001	Up	2.008	0.001	Up
*hsa-miR-6798-5p*	2.049	0.015	Up	1.866	0.019	Up	2.041	4.32E-04	Up
*hsa-miR-4419a*	1.737	0.013	Up	1.717	0.006	Up	1.851	9.32E-05	Up
*hsa-miR-296-3p*	1.811	0.031	Up	2.209	0.012	Up	1.726	0.109	Up
*hsa-miR-1227-5p*	1.549	0.013	Up	1.629	0.039	Up	1.653	0.147	Up
*hsa-miR-4656*	1.908	0.005	Up	1.617	0.025	Up	1.232	0.459	Up
*hsa-miR-3064-3p*	1.765	0.037	Down	1.529	0.001	Down	1.033	0.738	Down

*Abbreviations*: *SA* stable angina, *NSTE-ACS* non-ST segment elevation acute coronary syndrome, *STEMI* ST segment elevation acute myocardial infarction

### Gene ontology (GO) analysis

**Fig. 2:**
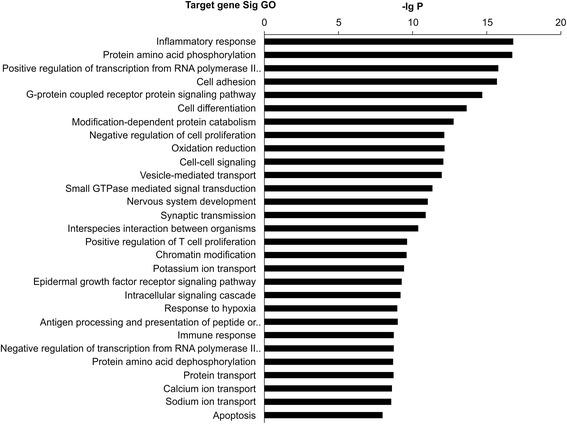
Gene ontology (GO) analysis

Differentially expressed miRNAs in patients without CAD and with SA or NSTE-ACS (compared with that in patients with STEMI) were found to be involved in inflammation, protein phosphorylation, RNA polymerase II-dependent transcription, cell adhesion, and other biological processes. Among these, inflammation, cell adhesion, T-cell proliferation, calcium transfer, and apoptosis were closely related to atherosclerosis (Fig. 2).

### Pathway analysis

**Fig. 3:**
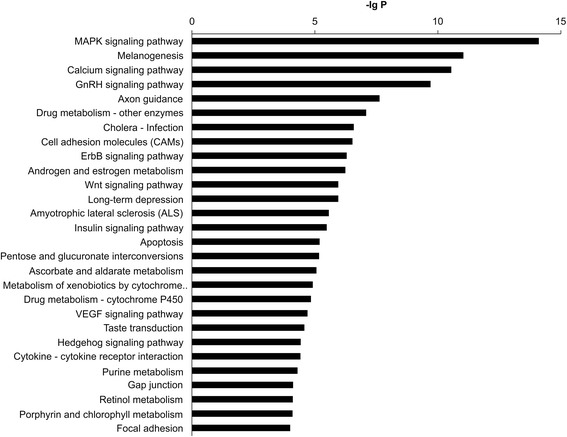
Pathway analysis

The KEGG database was used to analyze target genes of differentially expressed miRNAs. Differentially expressed miRNAs in control and in patients with SA or NSTE-ACS compared with that in patients with STEMI were found to regulate biological processes such as mitogen-activated protein kinase (MAPK) signaling, tumorigenesis, and calcium ion signaling. Among the identified cell signaling pathways, we identified the following pathways involved in the pathological process of atherosclerosis: adhesion molecules, ErbB signaling, metabolism, Wnt signaling, insulin signaling, apoptosis, vascular endothelial growth factor signaling, and cell factor receptor interactions (Fig. 3).

**Fig. 4:**
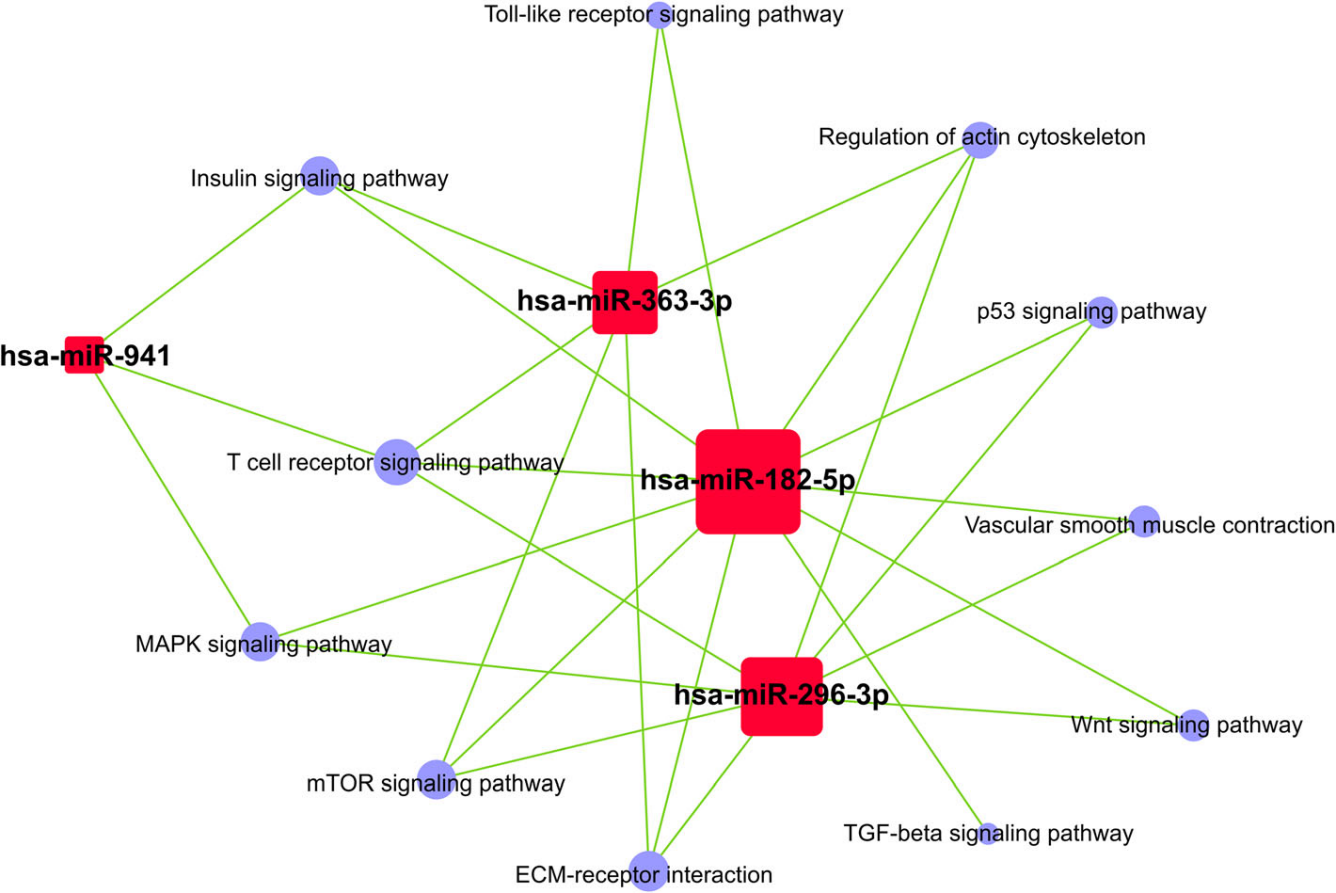
Microarray pathway network. The red rectangles are miRNAs, the purple circle are pathways, and the green lines represented the regulatory connections between miRNAs and pathways

miRNA-pathway network analysis showed that *miR-941* can participate in regulation of T-cell receptor signaling, insulin signaling, and MAPK signaling. Additionally, *miR-182-5p* was related to vascular smooth muscle cell constriction and mammalian target of rapamycin (mTOR) signaling. *miR-363-3p* was involved in Toll-like receptor signaling and actin cytoskeleton regulation. Bioinformatics functional predictions showed that the differential expressed miRNAs could be related to the pathological process of cardiovascular disease (Fig. 4).

### qRT-PCR

**Fig. 5:**
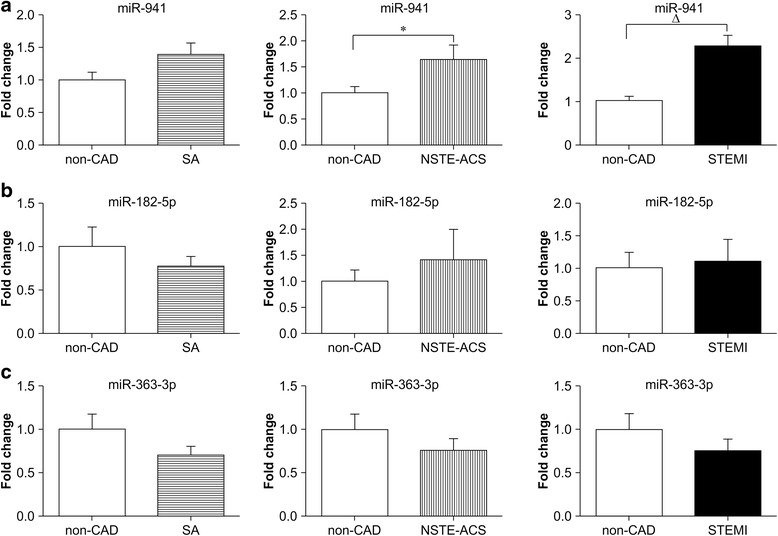
Expression profiles of candidate miRNAs in patients with and without CAD. The three candidate miRNAs were evaluated with qRT-PCR using 72 samples, including 16 non-CAD, 20 SA, 18 NSTE-ACS, and 18 STEMI samples. *miR-941* was significantly upregulated in the SA, NSTE-ACS, and STEMI groups compared with that in non-CAD patients (**a**). *miR-182-5p* (**b**) and *miR-363-3p* (**c**) were not significantly different in patients with CAD (SA, NSTE-ACS, and STEMI) compared with that in patients without CAD. Abbreviations: CAD: coronary artery disease, SA: stable angina, NSTE-ACS: non-ST elevation acute coronary syndromes, STEMI: ST elevation myocardial infarction. *P < 0.05, ^Δ^ *P* < 0.001

**Fig. 6:**
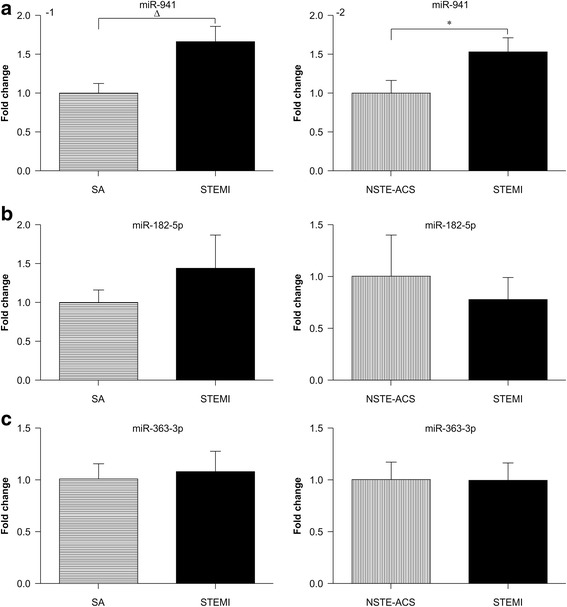
Expression profiles of candidate miRNAs in patients with SA, NSTE-ACS, and STEMI. The three candidate miRNAs were evaluated by qRT-PCR using 56 samples (20 cases of SA, 18 cases of NSTE-ACS, and 18 cases of STEMI). (**a**) *miR-941*, (**b**) *miR-182-5p*, and (**c**) *miR-363-3p* are shown. Abbreviations: CAD, coronary artery disease; SA, stable angina; NSTE-ACS, non-ST elevation acute coronary syndrome; STEMI, ST elevation myocardial infarction. *P < 0.05, ^Δ^ *P* < 0.001

Next, we performed qRT-PCR to verify the different expression of candidated gene like, *miR-941*, *miR-182-5p*, and *miR-363-3p*.The results showed that *miR-941* expression has no significant difference between the control patients (without CAD) and SA group. However, comparing with control patients, *miR-941* expression was significantly increased by 1.64- and 2.28-fold in patients with NSTE-ACS and STEMI, respectively, (*P* < 0.05; Fig. 5a). There were no significant differences in *miR-182-5p* and *miR-363-3p* among groups (*P* > 0.05; Fig. 5b and c). Additionally, the expression of *miR-941* was increased by 1.66-fold in patients with STEMI compared with that in patients with SA (*P* < 0.001; Fig. 6a-1) and by 1.52-fold in patients with STEMI compared with that in patients with NSTE-ACS (*P* < 0.05; Fig. 6a-2). There were no significant differences in *miR-182-5p* and *miR-363-3p* (*P* > 0.05; Fig. 6b and c).

**Fig. 7:**
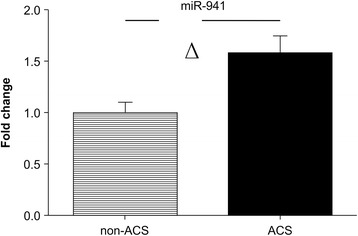
Expression profiles of *miR-941* in patients with and without ACS. *miR-941* expression was evaluated by qRT-PCR using 72 samples (36 patients without ACS and 36 patients with ACS). Abbreviations: ACS, acute coronary syndrome. ^Δ^ *P* < 0.01

Since there were no significant differences in *miR-941* expression between patients without CAD and patients with SA, we combined the two groups into the non-ACS group. Compared with that in the non-ACS group, the expression of *miR-941* was upregulated in the ACS group (FC: 1.62; *P* < 0.01; Fig. 7). NSTE-ACS and STEMI can reflect the degree of disease progression, and *miR-941* was significantly upregulated in patients with STEMI compared with that in patients in the non-ACS group (FC: 1.52; *P* < 0.05; Fig. 6a-2). The differential expression of *miR-941* in the two groups showed that the expression of *miR-941* was associated with the severity of ACS.

### Receiver operating characteristic (ROC) curve analysis

**Fig. 8:**
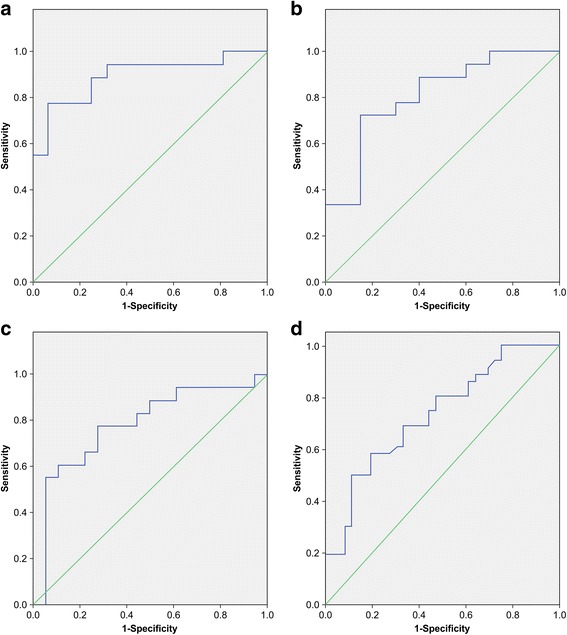
ROC curve analysis. **a** Expression profiles of *miR-941* in patients with STEMI and without CAD. **b** Expression profiles of *miR-941* in patients with STEMI and SA. **c** Expression profiles of *miR-941* in patients with STEMI and NSTE-ACS. **d** Expression profiles of *miR-941* in patients with and without ACS

Next, we performed ROC curve analysis to test the reliability of *miR-941* as a diagnostic biomarker of STEMI and ACS. Comparing with patients with STEMI, areas under the ROC curves were 0.896, 0.808, and 0.781 for patients in the control, SA, and NSTE-ACS groups, respectively; Comparing with patients in ACS group,the ROC curves of non-ACS group was 0.734 (Fig. 8). Thus, *miR-941* may act as a reliable biomarker of ACS, particularly STEMI. The area under the curve (AUC), 95% confidence intervals (CIs), and *p* values are summarized in Table 4.

**Table 4:** ROC curve analysis of *miR-941* expression in patients with ACS and STEMI

*miR-941*	AUC	95% CI	*P* value
Non-ACS versus ACS	0.734	0.619–0.848	0.001
Without CAD versus STEMI	0.896	0.779–1.000	0.000
SA versus STEMI	0.808	0.670–0.947	0.001
NSTE-ACS versus STEMI	0.781	0.622–0.939	0.004

*Abbreviations*: *SA* stable angina, *ACS* acute coronary syndrome, *NSTE-ACS* non-ST segment elevation acute coronary syndrome, *STEMI* ST segment elevation acute myocardial infarction, *CAD* coronary artery disease, *ROC* receiver operating characteristic, *AUC* area under the curve

## Discussion

Aberrant miRNA expression has been involved with a number of human diseases, including cardiovascular diseases [11, 21–23]. ACS has become a major public health problem owing to its high mortality and morbidity [24–26]. Thus, there is an urgent need to identify new diagnostic and therapeutic biomarkers of ACS; miRNAs may have such applications.

MiRNAs are involved in endothelial dysfunction, inflammation, apoptosis, angiogenesis, atherosclerosis, and other pathological processes involved in cardiovascular diseases [6, 27, 28], and miRNAs of circulartory system may be potential biomarkers of these diseases [7, 29]. *miR-135a*, *miR-31*, *miR-378*, and *miR-147* are biological markers of stable coronary heart disease [30]; *miR-1*, *miR-126*, and *miR-133a* have potential value in the diagnosis of UA [31]; and *miR-208b*, *miR-499*, and *miR-1* play a key role in the diagnosis, progression, and prognosis of acute myocardial infarction (AMI) [15, 32–34]. However, it is unclear whether miRNAs are differentially expressed with changes in the severity of coronary heart disease, including coronary stenosis and myocardial damage. Our findings suggested that *miR-363-3p*, *miR-941*, and *miR-182-5p* were differentially expressed among groups of patients with various types and degrees of coronary heart disease. Additionally, GO and KEGG pathway analysis showed that the differentially expressed miRNAs were involved in inflammatory responses, immune responses, MAPK signal, calcium pathway, ErbB signaling, and other cellular processes. In particular, MAPK signaling and immune responses are involved in the pathogenesis of atherosclerosis and affect the stability of plaques and the formation of blood clots. Furthermore, qRT-PCR analysis verified that *miR-941* was differentially expressed in plasma from patients with CADs (SA, NSTE-ACS, and STEMI) compared with that in patients without CAD. Thus, these data suggested that *miR-941* may have applications as a biomarker of CAD, with the ability to distinguish among ACS and non-ACS groups and to distinguish STEMI from SA and NSTE-ACS. Notably, *miR-941* was gradually upregulated as the degree of coronary artery stenosis and myocardial injury increased (SA < NSTE-ACS < STEMI); thus, *miR-941* was closely associated with the severity of ACS. Accordingly, we concluded that *miR-941* was a biomarker of ACS, particularly for STEMI, and could predict the severity and progression of coronary heart disease.

Recent bioinformatics analyses have shown that *miR-941* is involved in Wnt signaling, transforming growth factor (TGF)-β signaling, and insulin signaling [35, 36]. However, no research has been reported the role of *miR-941* in atherosclerosis and coronary heart disease. In our study, we found that *miR-941* may be associated with metabolism, inflammation, cell proliferation, and other biological processes through regulation of components involved in insulin signaling, MAPK signaling, T-cell receptor signaling, and other related pathways. However, we were not able to confirm the direct relationship between *miR-941* and the pathogenesis of atherosclerosis. In vitro and in vivo experiments are needed to clarify the biological function of *miR-941* in the pathophysiology of atherosclerosis and to determine whether *miR-941* is involved in pathological processes of inflammation, immunity, metabolism, and platelet activation. Although we found that *miR-941* was differentially expressed between ACS and non-ACS groups, additional studies are needed to establish a rapid, inexpensive method for miRNA analysis. Additionally, greater sample sizes are needed to further confirm the potential applications of this miRNA as a promising diagnostic tool for diagnosis of ACS.

## Conclusions

*MiR-941* was relatively higher in patients with ACS and STEMI, and could predict the severity and progression of coronary heart disease. Thus, *miR-941* may be a potential biomarker of ACS or STEMI.

## Additional files

Additional file 1: The mass detection report of samples. (DOCX 136 kb)

Additional file 1: Baseline Table 1:Original data of Baseline clinical characteristics of different groups used in microarray analysis. (XLSX 10 kb)

Additional file 3: Baseline Table 2:Original data of Baseline clinical characteristics of different groups used in qRT-PCR analysis. (XLSX 11 kb)

Additional file 4: MiRNA-gene-network and miRNA-targets-relation. (XLSX 382 kb)

Additional file 5: Microarray pathway network: Pathway-miRNA-relation and microarray pathway network. The red rectangles are miRNAs, the purple circle are pathways, and the green lines represented the regulatory connections between miRNAs and pathways. (XLSX 137 kb)

Additional file 6: Original data of Table 3: Differentially expressed miRNAs in microarray analysis. (XLSX 19 kb)

Additional file 7: Original data of Fig. 5a: MiR-941 was significantly upregulated in the SA, NSTE-ACS, and STEMI groups compared with that in non-CAD patients. (XLSX 14 kb)

Additional file 8: Original data of Fig. 5b: MiR-182-5p were not significantly different in patients with CAD (SA, NSTE-ACS, and STEMI) compared with that in patients without CAD. (XLSX 17 kb)

Additional file 9: Original data of Fig. 5c: MiR-363-3p were not significantly different in patients with CAD (SA, NSTE-ACS, and STEMI) compared with that in patients without CAD. (XLSX 17 kb)

Additional file 10: Original data of Fig. 6a-1: MiR-941 Expression profiles of candidate miRNAs in patients with SA, NSTE-ACS, and STEMI. The three candidate miRNAs were evaluated by qRT-PCR using 56 samples. (XLSX 13 kb)

Additional file 11: Original data of Fig. 6a-2: MiR-941 Expression profiles of candidate miRNAs in patients with SA, NSTE-ACS, and STEMI. The three candidate miRNAs were evaluated by qRT-PCR using 56 samples. (XLSX 13 kb)

Additional file 12: Original data of Fig. 6b: MiR-182-5p Expression profiles of candidate miRNAs in patients with SA, NSTE-ACS, and STEMI. The three candidate miRNAs were evaluated by qRT-PCR using 56 samples. (XLSX 15 kb)

Additional file 13: Original data of Fig. 6c:MiR-363-3p Expression profiles of candidate miRNAs in patients with SA, NSTE-ACS, and STEMI. The three candidate miRNAs were evaluated by qRT-PCR using 56 samples. (XLSX 15 kb)

Additional file 14: Original data of Fig. 7: Expression profiles of miR-941 in patients with and without ACS. (XLSX 15 kb)

Additional file 15: Table 4-ROC curve: Original data of ROC curve analysis of miR-941 expression in patients with ACS and STEMI. (XLSX 12 kb)

## Abbreviations

3′-UTR: 3′-untranslated region
ACS: Acute coronary syndrome
AMI: Acute myocardial infarction
AUC: Area under the curve
CI: Confidence interval
ECG: Electrocardiogram
FC: Fold-change
GO: Gene ontology
MAPK: Mitogen-activated protein kinase
MiRNA: MicroRNA
MTOR: Mammalian target of rapamycin
NSTE-ACS: Non-ST elevation ACS
qRT-PCR: Quantitative real-time reverse transcription polymerase chain reaction
ROC: Receiver operating characteristic
SA: Stable angina
STEMI: ST-segment elevation myocardial infarction
TGF: Transforming growth factor
UA: Unstable angina pectoris
