# Supplementary material for: miR-941 as a promising biomarker for acute coronary syndrome
Source: BMC Cardiovasc Disord. 2017 Aug 22;17:227. doi: 10.1186/s12872-017-0653-8 (PMC5568367; doi:10.1186/s12872-017-0653-8)
Supplement: Supplementary file 1 — The mass detection report of samples. (DOCX 136 kb) [file 12872_2017_653_MOESM1_ESM.docx]

| **The mass detection report of samples** | | | | | | | | | |
| --- | --- | --- | --- | --- | --- | --- | --- | --- | --- |
| Project name | | Affy miRNA chip | | | | | | | |
| The detection method of samples | | | | | | | | | |
| Extraction type | | ■RNA | | | | | | | |
| Extraction method | | TRIZOL | | | | | | | |
| Mass detction method | | ■NanoDrop ■ Agilent 2100 □ Qubit □Agrose Gel | | | | | | | |
| 样品检测结果 | | | | | | | | | |
| Number | Number of sample | concentration（μg/μl） | A260/280 | A260/230 | volume (μl) | aggregate（μg） | 2100 | | Result |
|  |  |  |  |  |  |  | 28S/18S | RIN |  |
| 1 | LHJ 3-2 | 0.1083 | 1.93 | 0.71 | 40 | 4 | 2.0 | 7.7 | A |
| 2 | XYQ 2-5 | 0.1042 | 1.91 | 0.73 | 40 | 4 | 1.3 | 9.5 | A |
| 3 | CJB | 0.1460 | 1.93 | 0.75 | 40 | 6 | 1.8 | 10.0 | A |
| 4 | GGC 2-4 | 0.1170 | 1.96 | 0.85 | 40 | 5 | 1.4 | 9.8 | A |
| 5 | GJJ 3-1 | 0.1087 | 1.92 | 0.69 | 40 | 4 | 1.4 | 9.6 | A |
| 6 | ZKX 2-1 | 0.1320 | 1.91 | 0.81 | 40 | 5 | 1.6 | 9.7 | A |
| 7 | YJZ 2-2 | 0.0933 | 1.89 | 0.60 | 40 | 4 | 2.3 | 7.7 | A |
| 8 | LJ 2-3 | 0.1439 | 1.92 | 0.68 | 40 | 6 | 1.6 | 9.8 | A |
| 9 | ZQF 2-5 | 0.1018 | 1.91 | 0.74 | 40 | 4 | 1.4 | 9.4 | A |
| 10 | SBH 2-5 SBH | 0.1876 | 1.96 | 1.19 | 40 | 8 | 1.6 | 9.9 | A |
| 11 | SYM | 0.1719 | 1.93 | 0.72 | 40 | 7 | 1.5 | 10.0 | A |
| 12 | YJJ | 0.2060 | 1.87 | 0.48 | 40 | 8 | 1.4 | 9.8 | A |
| 13 | PMZ | 0.1307 | 1.92 | 0.99 | 40 | 5 | 1.6 | 9.5 | A |
| 14 | MDK | 0.1381 | 1.89 | 0.57 | 40 | 6 | 1.5 | 9.7 | A |
| 15 | LRM | 0.1806 | 1.93 | 0.71 | 40 | 7 | 1.3 | 8.6 | A |
| 16 | DHZ ST 2 | 0.2269 | 1.94 | 0.69 | 40 | 9 | 1.5 | 9.7 | A |
| 17 | WSL ST | 0.2477 | 1.94 | 0.72 | 40 | 10 | 1.4 | 9.4 | A |
| 18 | WHQ | 0.1926 | 1.93 | 0.74 | 40 | 8 | 1.7 | 9.9 | A |
| 19 | HQT | 0.1451 | 1.85 | 0.47 | 40 | 6 | 1.7 | 8.0 | A |
| 20 | WG STE | 0.1509 | 1.95 | 1.04 | 40 | 6 | 1.5 | 9.7 | A |
|  | | | | | | | | | |
| 样本检测图谱 | | | | | | | | | |
| 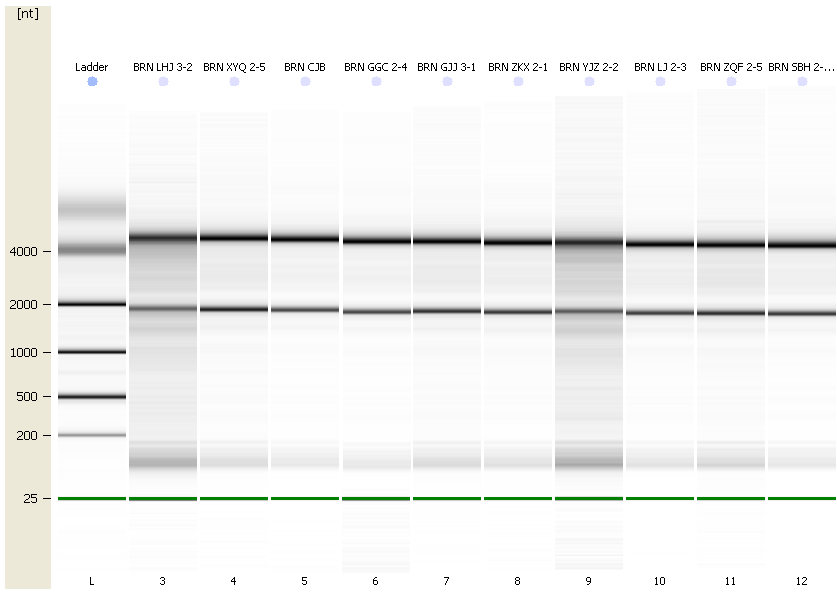  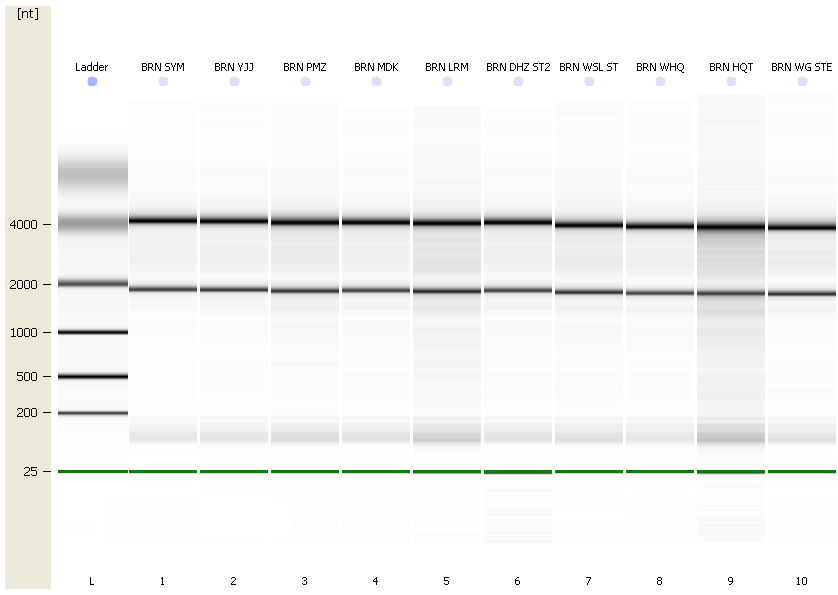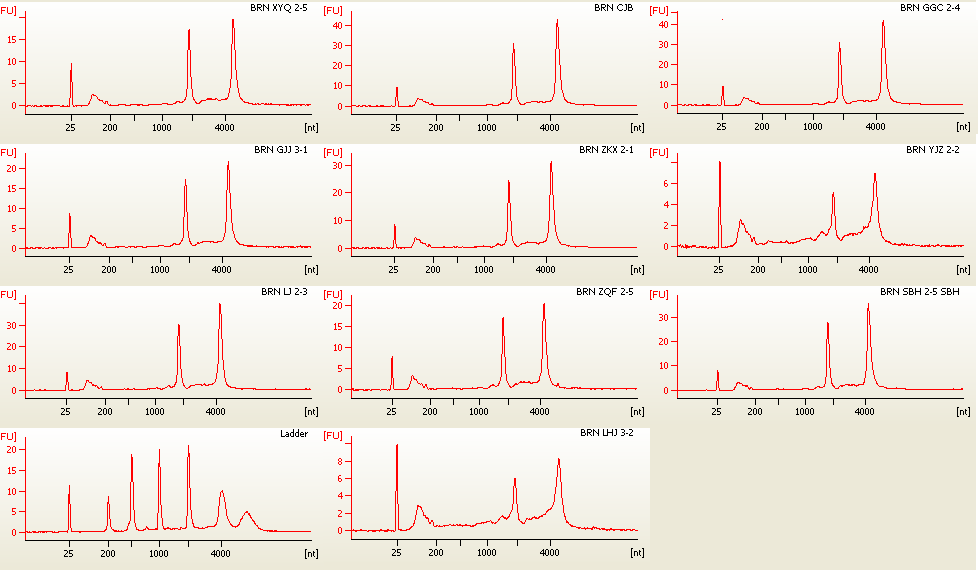  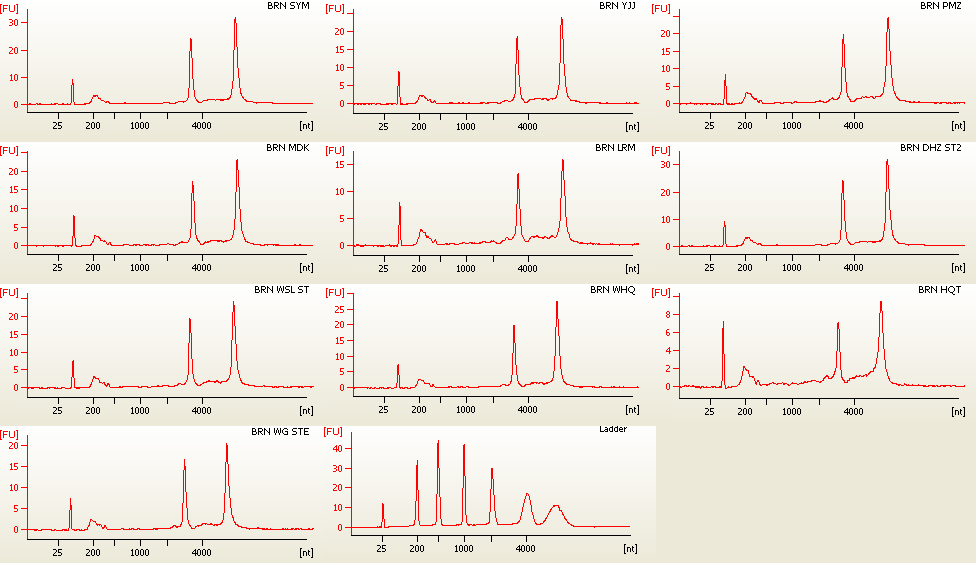 | | | | | | | | | |
| RNA quality judgment standards: RNA (including the miRNA)  Class A: quality satisfies the requirement of experiment (RIN > = 7 and 28 s/s > = 0.7), and total amount can satisfy for more than 2 experiments  Class B: quality satisfies the requirement of experiment (RIN > = 7 and 28 s/s > = 0.7), and total amount can satisfy for less than two experiments  Class C：the quality is not fully meet the requirements of the experiment (RIN > = 7 and 28 s/s < 0.7 or 6.0 = < RIN < 7.0, the mycoplasma or bacterial contamination), has the certain risk  Class D: the quality meet the experimental requirements (RIN > = 7 and 28 s/s > = 0.7), but does not meet the total one experiment E: quality did not meet the requirements of the experiment (RIN (6), it is not recommended for subsequent experiment | | | | | | | | | |
